# Instant Heart Failure Diagnosis Using Point-of-Care Ultrasound (POCUS) in a Patient Incorrectly Diagnosed With Asthma

**DOI:** 10.7759/cureus.27456

**Published:** 2022-07-29

**Authors:** Larry Istrail

**Affiliations:** 1 Hospital Medicine, Inova Fairfax Hospital, Falls Church, USA

**Keywords:** sob - shortness of breath, lung ultrasound (lus), 2d echo, heart failure with reduced ejection fraction, pocus (point of care ultrasound

## Abstract

Congestive heart failure is one of the most common causes of hospitalization in the United States, yet it often goes undetected due to the poor sensitivity of chest X-rays for detecting pulmonary edema. In this case, a patient presented with one year of shortness of breath and a diagnosis of asthma; however, a three-minute point-of-care ultrasound (POCUS) exam revealed that the correct diagnosis was in fact congestive heart failure. This highlights the importance of incorporating POCUS into the physical exam of any patient presenting with cardiopulmonary symptoms.

## Introduction

Congestive heart failure is a leading cause of hospitalization in the United States [1]. However, in up to 20% of cases, the condition goes undetected in patients at urgent care or primary care visits even when signs and symptoms are present [2]. Chest X-ray is the standard first-line choice of imaging; however, it is not sensitive or specific enough to diagnose pulmonary edema reliably [3]. In contrast, point-of-care ultrasound (POCUS) is a diagnostic tool that is much more sensitive and specific [4-6], and therefore more appropriate for screening patients with cardiopulmonary-related symptoms.

## Case presentation

A 50-year-old female with a history of type 2 diabetes and asthma presented to the hospital with one year of shortness of breath that had acutely worsened in the weeks prior to the presentation. She had visited multiple urgent care centers where a chest X-ray or EKG had been performed. At the initial visit, the chest X-ray was unremarkable and her electrocardiogram showed a left bundle branch block. She was diagnosed with an asthma exacerbation and prescribed an inhaler, which did not improve her symptoms. She was seen weeks later at another urgent care, and a chest X-ray was done to evaluate her ongoing dyspnea. She was diagnosed with pneumonia and prescribed a course of azithromycin, which also failed to improve her symptoms.

**Figure 1:**
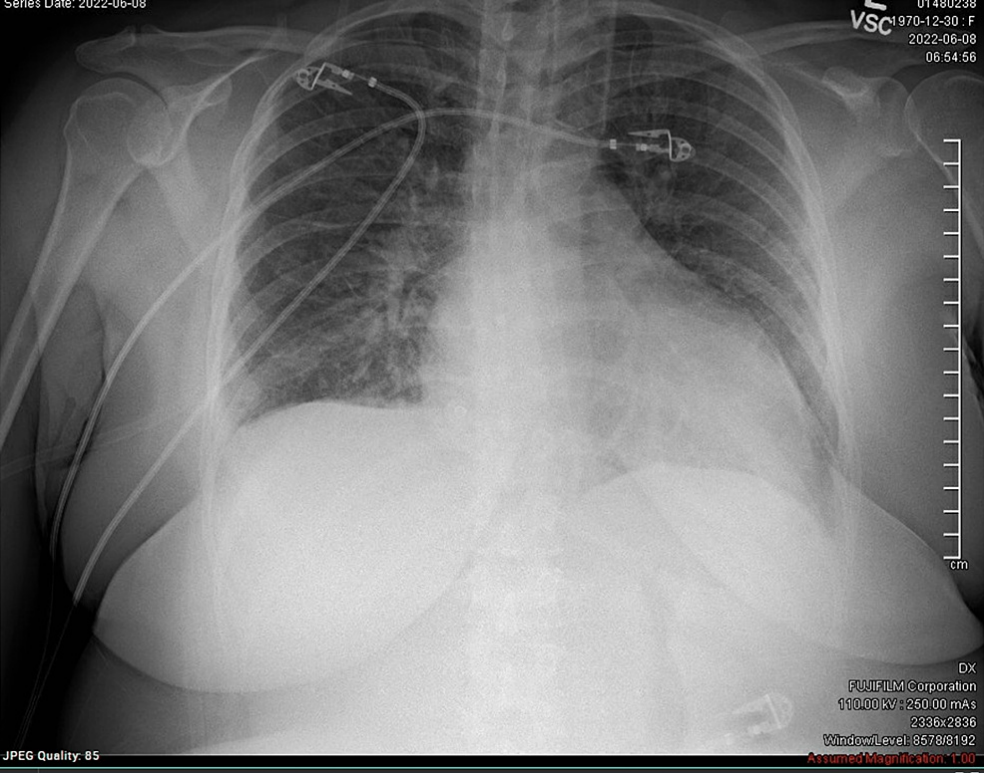
AP chest X-ray on arrival at the emergency department The X-ray was read as mild right basilar atelectasis with possible vascular congestion

Her shortness of breath persisted and she presented to the emergency department. On arrival, she was afebrile, tachycardic to 105 bpm, and breathing at 20 times per minute with oxygen saturation of 100% on ambient air. A chest X-ray was read by the radiologist as right basilar atelectasis and possible vascular congestion (Figure 1).

The patient was treated with an albuterol nebulizer and intravenous corticosteroids for presumed asthma exacerbation given her known history of asthma.

Shortly after, the patient was admitted to the hospital and evaluated by the author with POCUS. With the head of the bed at 45 degrees, the jugular vein was assessed just superior to the clavicle with the ultrasound probe in the transverse orientation. The jugular vein was noted to be distended without venous pulsations, consistent with jugular venous distention (Video 1).

**Video 1:** Jugular venous distention (JVD) seen with POCUS The top vessel is the right internal jugular vein, above the internal carotid artery. It is distended without jugular venous pulsations present with the patient at 45 degrees, consistent with jugular venous distention (JVD) POCUS: point-of-care ultrasound

Sonographic examination of the lungs revealed regular pleural lines with lung sliding intact and lung rockets in multiple lung fields bilaterally (Video 2).

**Video 2:** Lung rockets (3 or more B-lines) in a rib space seen with POCUS in pulmonary edema The pleural line is smooth and lung sliding is present. There are multiple vertical projections from the pleura. Lung rockets are present in multiple rib spaces POCUS: point-of-care ultrasound

The base of the lungs revealed bilateral pleural effusions with compression atelectasis (Video 3).

**Video 3:** Right pleural effusion with compression atelectasis The hypoechoic space above the diaphragm is a pleural effusion that is applying external compression to the lung base, causing atelectasis

A parasternal long-axis view of the heart was obtained, and upon visual inspection, it was confirmed that the patient had globally reduced systolic function (Video 4).

**Video 4:** Reduced ejection fraction observed in parasternal long axis view with POCUS POCUS: point-of-care ultrasound

The IV steroids and nebulizer treatments were held, and she was started on IV furosemide with significant improvement in her symptoms. An echocardiogram was done, which confirmed the POCUS findings of a reduced ejection fraction. She underwent a left heart catheterization, which revealed non-obstructive coronary artery disease. During her hospitalization, she diuresed five liters, her weight decreased, and her shortness of breath and orthopnea resolved. She was started on appropriate guideline-directed medical therapy and discharged with close cardiology follow-up.

## Discussion

Shortness of breath is one of the most common reasons for seeking medical care, yet its differential diagnosis can be quite broad. In this case, the patient's history of asthma and nondiagnostic chest X-rays resulted in significant patient morbidity through incorrect diagnoses, incorrect treatments, and a lack of improvement in her symptoms for one year. Chest X-rays have poor sensitivity for detecting pulmonary edema [3], can only detect pleural effusions greater than 300 milliliters [7], and often cannot differentiate atelectasis from pneumonia. In contrast, POCUS has nearly perfect diagnostic accuracy for pulmonary edema [4,5,8] and other conditions that distort the pleura. Lung ultrasound can detect pleural effusions of less than 10 milliliters [9], and is 100% sensitive and specific for effusions over 100 milliliters [10].

In this case, the correct diagnosis was made in under three minutes. The volume status was obtained using jugular venous ultrasound protocols described previously [11]. Jugular venous distention was present above the clavicle with the head of the patient's bed at 45 degrees, confirming an elevated right atrial pressure.

Lung ultrasound was performed to evaluate for causes of dyspnea by evaluating for lung sliding, A-lines, or B-lines. In this patient, the pleural lines were smooth and emitted vertical projections (B-lines) in multiple rib spaces, termed "lung rockets," which is nearly diagnostic of pulmonary edema [8]. These diffuse lung rockets were corroborated by the presence of bilateral small pleural effusions and further corroborated by a qualitatively reduced ejection fraction as seen in the parasternal long-axis view.

## Conclusions

As demonstrated in this case, morbidity can be substantially prevented if POCUS is incorporated into the initial evaluation of any patient presenting with shortness of breath. A physical exam and a chest X-ray are often not sufficiently sensitive to diagnose pulmonary edema or other cardiopulmonary pathologies. POCUS offers a unique ability to incorporate a more thorough evaluation of the venous system, the lungs, and the heart into the patient's initial examination to obtain a more accurate diagnosis.
